# Towards an eco-social circular economy: exploring the feasibility study of pyrolysis on agricultural feedstocks

**DOI:** 10.1007/s13399-024-06361-z

**Published:** 2024-12-06

**Authors:** Thomas Allison, Kumar Vijayalakshmi Shivaprasad, Abdullah Malik, Rehman Rafiq, Yaodong Wang, Anthony Paul Roskilly

**Affiliations:** 1 Department of Engineering, Durham University, Durham, DH1 3LE UK; 2 Hybrid Gasification Ltd, Newcastle Upon Tyne, NE15 6UN UK

**Keywords:** Agriculture, Auger, Barley, Bean, Lower heating value, Oil seed rape, Pyrolysis, Wheat

## Abstract

The agricultural sector is challenging to decarbonise due to its reliance on heavy machinery and fossil fuels, which face issues when decarbonising via methods such as electrification. However, agriculture provides opportunities to generate renewable energy via biomass sources due to their abundance within this sector. This feasibility study used a continuous auger pyrolysis system to assess how straw waste from a medium-scale arable farm could convert energy from an external electrical source into usable chemical potential. Wheat, barley, oil seed rape (OSR), and bean straw have all been processed and pyrolysed under different temperatures and auger feed rates. The syngas product was then analysed, considering its composition and the lower heating value. Results indicate that the percentage of carbon monoxide and hydrogen and the total volume of syngas increased with temperature. In addition, the syngas’ energy quantity increased despite the product’s decreasing heating value. The case study’s annual energy demand was equal to 14.4% of the 3900 GJ maximum potential contained within the syngas, and thus it can be concluded that there is potential for the application of this system towards a circular economy. The system’s cold gas, net, and electrical conversion efficiency were also assessed with maximum values of 37.1%, 30.1%, and 174.4%, respectively. Furthermore, the statistical analysis confirms high predictability for wheat, barley, bean, and OSR feedstocks, with a general linear model showing high accuracy across all.

## Introduction

Developing net-zero technologies is paramount if the United Kingdom (UK) is to meet its net-zero target of 2050 [1]. Such a commitment will require a massive overhaul of the energy network, transitioning away from the use of fossil fuels, and protecting against the cost of energy spiking. Utilising UK-based energy supplies would remove its reliance on other countries for its energy security, protecting against the volatility of the fossil fuel market, and making this transition from fossil fuels viable. In addition, unsustainable energy use has led to the exploitation of fossil fuels, leading to vast emissions of greenhouse gases and a global warming of 1.1 °C [2]. Despite this, emissions continue to increase. By utilising agricultural waste to help meet the energy demands of a farm, the emissions from the agricultural industry would be reduced, limiting the reliance on fossil fuels. Using green technologies alongside gasification techniques could allow for a carbon-negative process which provided the cultivation of the fuel source and crops and employed renewable fuels [3, 4]

The agricultural industry contributes to the UK’s total emissions by releasing carbon dioxide (1.7%), methane (48%), and nitrous oxide (69%), which occurs due to the use of agricultural soils, stationary combustion, and vehicular machinery [5]. Despite little progress towards net zero, it has been reported that 64% of farmers consider it important, with pyrolysis systems providing a potential solution. Pyrolysis supports farmers in reducing their carbon footprint, by allowing them to meet their stationary combustion needs (propane generators) as well as vehicular fuels, in a low-carbon way, aiding in moving the industry towards a net zero future. Pyrolysis also provides a potential for carbon sequestration by utilising the high carbon content of biochar and locking it into the soil, employing its fertilising properties to enrich the ground [3, 4].

The thermochemical process of pyrolysis is a proven technology, which has shown promise as a renewable energy resource. In pyrolysis, thermochemical decomposition of feedstock occurs when it is exposed to elevated temperatures in the absence of oxygen (an inert atmosphere) [6]. This causes high molecular vibrations which results in complex molecules breaking down into smaller constituents [7]. A variety of pyrolysis technologies exist, though this report specifically focuses on electrical pyrolysis which is mainly categorised into slow, fast, and flash pyrolysis and this categorisation depends on heating rate, temperature, and vapour residence time [6].

There are three main outputs of pyrolysis, biochar (solid), bio-oil (liquid), and bio-syngas (gas). Bio-syngas studies focus on the products’ composition alongside the oil and char. They compare syngas properties against several variables assessing how the composition changes [8, 9]. Investigations into bio-syngas have been conducted on a wide scale. Jun and his associates concentrated on hydrogen-rich syngas from municipal solid waste and wheat straw combinations, assessing the product distribution at pyrolysis temperatures between 500 and 1000 °C [10]. Other studies investigated how hydrogen yield varied with temperature, power, moisture content, and the effects of an additive [11, 12]; or allowed the modelling of lignin and hemicellulose ratios with product yield. Characteristics such as the effect of temperature, heating rate, and particle size were also considered in detail, allowing for the assessment of gas composition and product distributions [8, 9]. From this, it is clear that no research has focused on UK agricultural residues specifically or how the gas composition varies between agricultural feedstocks.

The optimisation of syngas composition depends on pyrolysis variables, allowing for ideal conditions to be determined. Literature found that a higher temperature resulted in a higher heating value being obtained for the syngas produced [13–15]. However, these results were presented for a low-temperature range and therefore may not fully represent the actual effects expected, particularly for the temperature ranges included by fast pyrolysis. Such criticism is backed up by the work of Fu and his research team who covered a higher temperature range and saw a minor decrease in heating value past 800 °C. From this, there is scope for further study to assess how the heating value of syngas produced changes under fast pyrolysis conditions and higher temperatures [16].

**Fig. 1:**
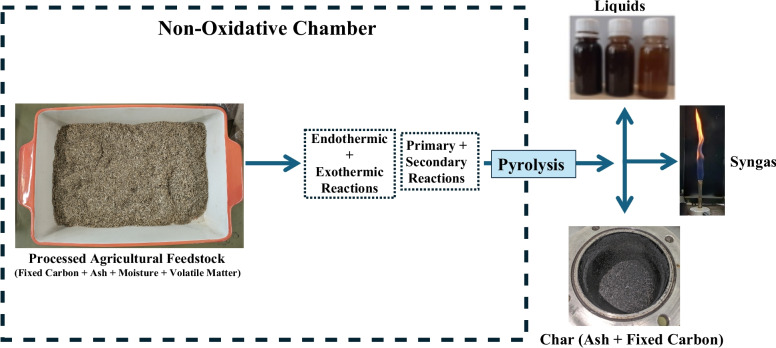
Overview of the study

Ultimately, the agricultural sector’s reliance on fossil fuels and the associated decarbonisation challenges related to heavy machinery present a clear issue that needs to be addressed. Therefore, a research gap exists due to the limited study of agricultural residues under fast pyrolysis using a continuous auger set-up, alongside a lack of data regarding the energy content of syngas produced. This method may support the decarbonisation of heavy machinery due to the production of syngas. Thus, the aim of this paper is to identify the optimum states under which to conduct fast pyrolysis using an electrically heated, continuous auger design, which is a novel technology due to the sole objective of maximising syngas production. The schematic representation of the overview of the study is depicted in Fig. 1. Therefore, as syngas is the primary product, there is a focus on maximising the energy potential within this. A medium-scale UK arable farm acted as a case study, providing biomass for the experiments, as well as a scale to compare energy production. Through this, the paper seeks to assess the feasibility of using such a pyrolysis set-up to provide a net zero energy supply to a UK farm, outlining an opportunity for the decarbonisation of agriculture, and demonstrating the energy potential of waste straw.

## Materials and methods

This work aims to assess the energy potential and characteristics of syngas produced using an electrically heated, auger-fed, pyrolysis system. It varies considerably from similar projects in literature by solely focusing on the syngas and maximising the energy content as well as considering different feedstocks and conditions to ensure it fills a clear research gap.

### Agricultural feedstock

The case study considered for this project is an arable farm in Cambridgeshire, UK. They supplied 4–5 kg samples of each crop from their 2022–2023 season, as seen in Table 1. Additionally, they provided estimates regarding their grain yields for the season in question, which allowed for an estimation of the waste available for a scaled-up analysis.

**Table 1:** Crop distribution (2022–2023) [17]

Crops	Land, ha	Straw/kg	Energy/GJ
Wheat	99	400,000	6930
Barley	59	160,000	2780
Oil seed rape (OSR)	31	50,000	870
Beans	13	40,000	680

The farm also supplied data regarding their energy use, consisting of electric bills, vehicle fuel volume, and liquid fuel for heating and drying purposes, as seen in Table 2. From this, the energy demand at the farm could provide a baseline comparison between the energy potentials of the syngas produced.

**Table 2:** Farm energy use (2022–2023) [18]

Energy	Electricity	Heating	Red diesel	White diesel
Amount/GJ	18.6	95.8	382.9	95.7

### Sample processing

**Fig. 2:**
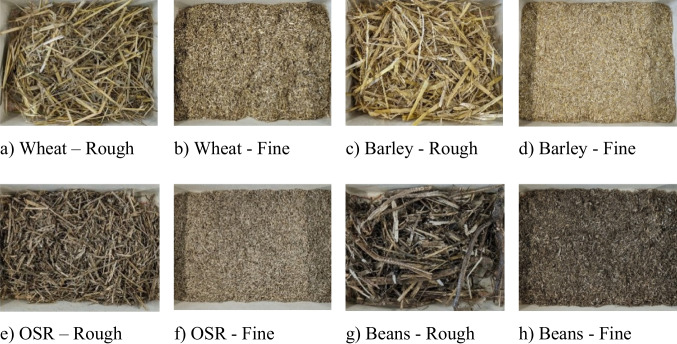
Straw types—before (rough) and after (fine) pre-processing—displayed at the same scale

The samples provided by the farm consist of the straw and stems from the crops under investigation, as seen in Table 1. For the straws to pass effectively through the pyrolyser, they needed to be processed to less than 10 mm in size, as seen in Fig. 2. This ensured that they would not be large enough to cause blockages and jam the auger in addition to maintaining a sufficient size to prevent clumping. Such issues had previously been caused using fine powdered biomass. To produce a suitable sample size, garden and paper shredders alongside a food processor were employed to process the straw.

Table 3 provides details of biomass properties. A proximate analysis specifies the elemental composition and provides the data on the percentage of moisture (M), volatile matter (VM), fixed carbon (FC), and ash (A) in the biomass. An ultimate analysis indicates the weighted percentage of carbon (C), hydrogen (H), and nitrogen (N) within the feedstock, and lignocellulosic analysis provides the details of three primary constituents of biomass such as cellulose (Ce), hemicellulose (He), and lignin (Li).

**Table 3:** Properties of biomass

Proximate analysis					Ultimate analysis			Lignocellulosic analysis		
Biomass	M	VM	FC	A	C	H	N	Ce	He	Li
Wheat	7	82	9	2	41.3	5.50	0.57	34.3	27.5	17.7
Barley	12	74	13	3	42.4	5.59	0.47	37.6	34.8	15.8
OSR	2	80	15	3	23.3	1.92	0.38	49.5	12.7	17.7
Beans	2	77	13	8	21.4	2.46	0.97	21.4	19.6	10.2

### Performance parameters

Process efficiency is an important consideration that must be quantified as it allows for an indication of the utility of the system. This is done in several ways depending on the focus of the study. All these values are useful indications of the energy requirements and therefore key factors in determining the feasibility of different designs and applications. This section provides the equations being used in this work.

#### Cold gas efficiency

Cold gas efficiency (CGE) assesses the energy potential in the syngas compared to the energy potential of the feedstock [19]. CGE is the ratio of the energy potential contained in the syngas output, compared to the energy potential of the feedstock supplied and it can be calculated by using Eq. (1):

1 $$CGE= \frac{{LHV}_{g}\left(\frac{MJ}{{Nm}^{3}}\right)\times V( \frac{{Nm}^{3}}{{kg}_{f}}) }{{LHV}_{f} (\frac{MJ}{{kg}_{f}})}$$

#### System’s net efficiency

Net efficiency considers the total energy input compared to the total energy output [19]. It is important to include the electrical energy input in the efficiency as the system being assessed for this report relies on electrical energy for heating. This is presented as part of the system’s net efficiency, as seen in Eq. 2:

2 $${\eta }_{NET}=\frac{{LHV}_{g}\left(\frac{MJ}{{Nm}^{3}}\right)\times V\left( \frac{{Nm}^{3}}{{kg}_{f}}\right)}{{LHV}_{f} \left(\frac{MJ}{{kg}_{f}}\right)+[{(P}_{heat}+{P}_{m})\left(\frac{MJ}{s}\right)\times {T}_{r} (\frac{s}{{kg}_{f}})]}$$

with ${P}_{heat}\left(\frac{MJ}{s}\right)$ and ${P}_{m}\left(\frac{MJ}{s}\right)$ relating to the power requirements of the heater and motor, respectively, in addition to ${T}_{r} (\frac{s}{{kg}_{f}})$ being the time required to pass 1 kg of feed through the auger.

#### Electrical efficiency

Electrical efficiency can be determined considering the useful energy required to operate the system and the quantity of syngas produced, defined in Eq. 3 [19]:

3 $${\eta }_{elec}=\frac{{LHV}_{g}\left(\frac{MJ}{{Nm}^{3}}\right)\times V\left( \frac{{Nm}^{3}}{{kg}_{f}}\right)}{[{(P}_{heat}+{P}_{m})\left(\frac{MJ}{s}\right)\times {T}_{r} (\frac{s}{{kg}_{f}})]}$$

### Experimental setup

**Fig. 3:**
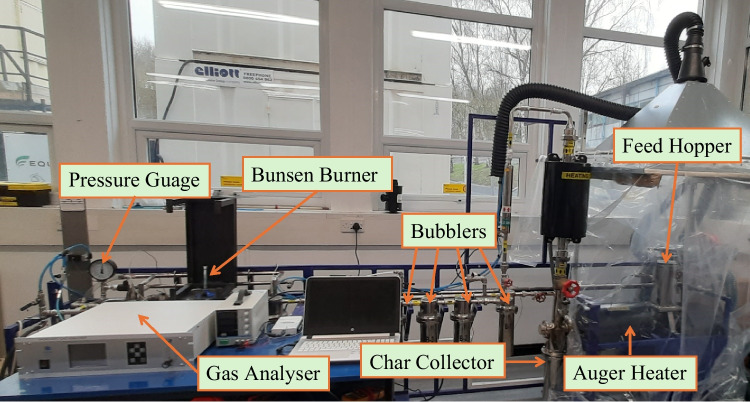
Photographic view of pyrolyser

The experimental set-up employed is shown in Fig. 3, which consists of several key components: char collector, auger heater, bubblers, feed hopper, gas analyser, pressure gauge, and Bunsen burner. Initially, the pyrolyser is heated to the desired temperature by an electric heater preset to maintain the temperature for a specified time duration. Once the system reached the set temperature, testing could commence. A hopper is used to deliver feedstock into the system which is subsequently sealed and secured throughout the test. Once the feedstock passed through the auger heater, the remaining mass was contained in a sealed stainless steel ash collector where it remained for the entire duration of that test. The vapourised products (syngas and tars/oils) are fed through three water-bubbling chambers that remove tars/oils leaving the clean syngas required. Syngas finally passed through two cooling coils and a filter before it reached the end of the pyrolyser.

### Methodology

The assessment of all four biomass types was key to the study, considering wheat, barley, OSR, and bean straws, as seen in Table 1. These were assessed to optimise conditions, maximising the energy potential of the syngas produced, and were run at temperatures of 750 °C, 800 °C, 850 °C, and 900 °C. In addition to temperature, the auger feed rate was assessed considering both 5 rpm and 10 rpm. The syngas produced was evaluated by the gas analyser, assessing the vol % of CO, CO_2_, H_2_, CH_4_, C_2_H_4_, C_n_H_m_, and N_2_.

## Results and discussion

### Syngas composition and volume

This section provides the composition and volume of syngas produced from all four feedstocks under variable temperature and feed rate conditions. Blue- and red-shaded bars represent the 5 rpm and 10 rpm results accordingly, with different shades and hash patterns relating to different temperatures. Lighter shades indicate higher temperatures.

#### Effect of temperature and auger feed rate

**Fig. 4:**
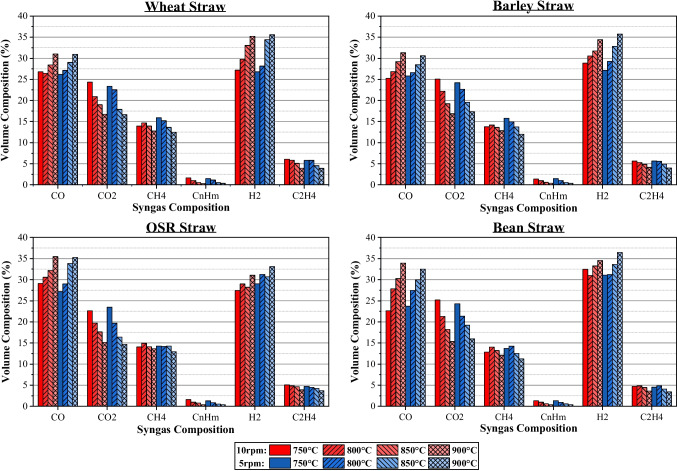
Syngas composition

The effect of operating temperature on syngas compositions is illustrated in Fig. 4. The general trends observed for the effects of temperature were that CO and H_2_ vol% increased, whereas the composition of CO_2_, CH_4_, C_n_H_m_, and C_2_H_4_ all decreased (though by varying amounts). This is expected as a higher temperature leads to greater degradation of complex molecules into simpler ones, confirming the results obtained by Luo et al. [13]. Syngas composition varies between feedstocks, though similar trends are present for all. Hydrogen volume for wheat and barley varied by 8% from the maximum to minimum temperatures. This contrasts with OSR and beans where a volume range of 3.5% and 5.5% was observed for the same temperature range. Additionally, beans display a significant vol% increase for CO, and a decrease in CO_2_, resulting in a 10% difference. Though similar trends were observed for the other feedstocks, a lower difference was visible, which may be attributed to variance in the composition of each feedstock. This change is explained by the ultimate, proximate, and lignocellulosic analysis results, presented in Table 3. From the proximate and ultimate analyses, it is clear that there is a greater quantity of H_2_ within wheat and barley, though this may be due to a greater level of moisture being present in those feedstocks. From this, it can be attributed that the change in the amount of H_2_ present in the feedstock causes the larger change in H_2_ volume seen in the syngas compositions. As the temperature increased, the decomposition of hemicellulose and lignin increased, producing more CO, confirmed by the results presented by Yang et al. [18]. The large change in CO seen for beans can be attributed to the lower quantities of cellulose, hemicellulose, and lignin present in this feedstock. From this, bean straw likely contains a greater quantity of extractives, which decompose into CO and CO_2_, explaining why the vol% of CO changes dramatically.

Changing the feed rate through the auger heating element had a smaller effect on the composition results compared to those seen for temperature. However, this is due to the smaller sample size observed. Decreasing the feed rate increased the amount of hydrogen obtained at both 900 °C and 850 °C but decreased the volume obtained at 800 °C and 750 °C. One solution to this could be that softer feedstock such as wheat and barley compressed more in the auger screw at a slower speed and therefore a more densely packed layer was exposed to heat. Dense layer might lead to a lower average heat exposure and therefore explains the lower quantity of H_2_ obtained. This was backed up by how the decrease was not observed to the same extent for OSR and beans. The feed rates obtained for these samples were significantly higher than wheat and barley, as seen in Table 4. Thus, it is theorised that these samples are denser and undergo limited compression. This leads to a consistent feed rate through the auger (with doubling auger speed more closely leading to double the mass passing through) and therefore the trends observed within the auger speeds more closely match those expected.

**Table 4:** Feed rate (kg/h)

Biomass	10 rpm	5 rpm
Wheat	0.410	0.289
Barley	0.334	0.229
OSR	0.825	0.493
Beans	0.998	0.528

#### Syngas volume

The total volume of the syngas could be determined by using the corrected flow rate and test duration. This could then be scaled up to identify the quantity of syngas possible from the entirety of the farm’s waste.

**Fig. 5:**
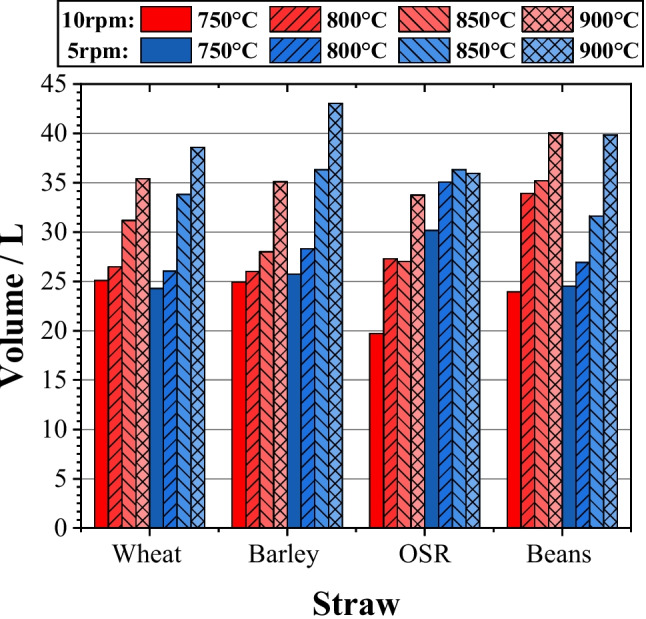
Syngas volume

From Fig. 5, an increase in temperature resulted in a greater volume of gas being obtained. This confirms the results presented by Xu and team who demonstrated that an increase in temperature increased syngas yield, due to a greater level of decomposition occurring at higher temperatures [14]. The volume obtained for different feedstocks varies; in particular, OSR produces lower gas volumes which can be attributed to the lower hemicellulose and lignin present within the feedstock, as shown in Table 3.

Slower speed (5 rpm) generally produced a larger volume of syngas due to the greater extent of heat exposure, resulting in a higher level of decomposition and therefore a greater volume of syngas. This appears to be the case for 900 °C but was not consistent among lower feed rates. The effect of density, as noted earlier, is the defining factor behind this result. At 5 rpm, a thicker layer tended to build up within the auger and therefore this outer layer acted as a barrier protecting the innermost layers from the full effects of heating. OSR does not appear to be affected, whereas beans displayed the opposite trend. Thus, it could be concluded that the composition of beans is likely to decompose at a lower heating rate compared to the composition of the other samples.

### Energy

#### Energy input

The system used for these experiments was an electrically heated auger reactor; therefore, the supply of electrical power to the heater is paramount. This was recorded, by the Invertor Resistive Heater DHC 6510R, as current and voltage data for each temperature increment. From this, the total power can then be quantified, with a maximum of 1 kW occurring under 900 °C.

Using the power data, the energy to run the system can be calculated. To do this, the feed rate data, shown in Table 4 was compared to the tonnage of straw available, allowing for the required system run time to be deduced. Hence, the laboratory set-up would require a continuous run time of 176 or 256 years at 10 rpm or 5 rpm, respectively. This would be unfeasible and demonstrates the requirement to scale up the system, for example, to a feed rate of 200 kg/h, reducing run time to 270 days. However, it should be noted that on larger systems, the energy required for heating as well as the syngas composition and the volume obtained would not scale linearly so thorough investigations would be needed before a real-world application was in place. This falls outside of the scope considered here and as a result, the calculations presented by this paper are based entirely on the laboratory system.

As depicted in Table 5, the energy required to run the system to process all feedstock through the pyrolyser ranged between 4000 and 8000 GJ under 750 °C at 10 rpm and 900 °C at 5 rpm, respectively. These values are displayed in GJ for ease of comparison between the farm’s demand, syngas produced, and energy requirements.

**Table 5:** Total energy input (GJ)

Temp. (°C)	Wheat		Barley		OSR		Beans	
	5 rpm	10 rpm	5 rpm	10 rpm	5 rpm	10 rpm	5 rpm	10 rpm
900	5050	3565	2562	1758	371	222	294	157
850	4455	3145	2260	1551	327	196	259	138
800	4112	87	2086	1431	302	181	239	128
750	3679	2597	1867	1281	270	162	214	114

### Energy output potential

A defining factor of this study was the LHV of the syngas, which refers to the amount of heat produced from the complete combustion of a unit of fuel, minus the latent heat of vaporisation of the water vapour produced during the combustion process.

#### Heating value

**Fig. 6:**
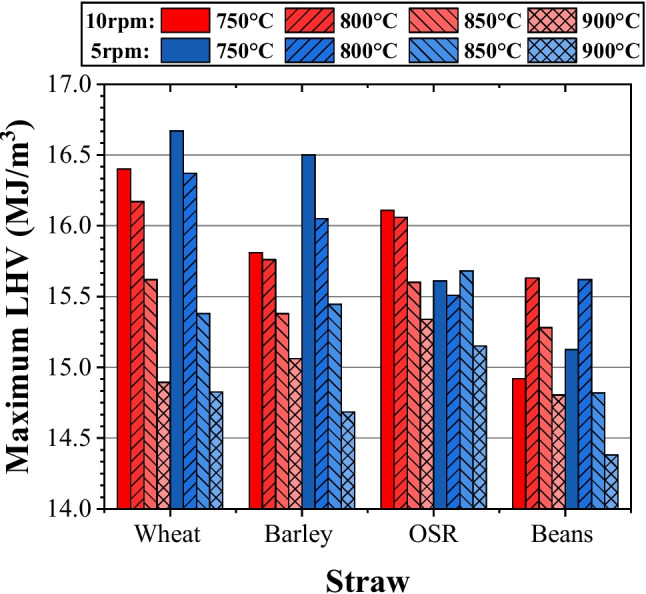
Syngas lower heating value

Figure 6 illustrates how the LHV of the syngas varies with feedstock, temperature, and feed rate. LHV decreases with temperature, with large differences being visible for wheat and barley, in comparison to OSR and bean samples. It is evident that for temperatures below 800 °C, an increase in temperature increased the heating value whereas over 800 °C, the heating value of the gas decreased. Such a result can be attributed to the high volume of hydrogen obtained which increased with temperature, contrasting with the results presented by Fu et al. [16]. From this, it is reasonable to assume that at temperatures above 800 °C, the LHV does decrease. Figure 5 also shows a decrease in LHV from 5 to 10 rpm feed rates at higher temperatures. Wheat and barley at 750 °C and 800 °C contradicted this, which can be attributed to the lower volume composition of H_2_ when running at 5 rpm under these temperatures. This was due to the limited exposure, of the feedstock, to heat caused by a thicker layer building up within the auger.

#### Total energy potential

**Fig. 7:**
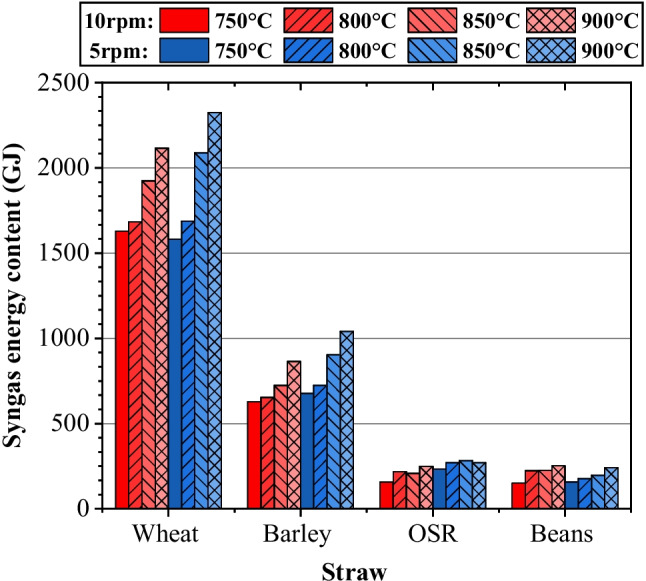
Syngas energy content (GJ)

Linking the volume estimations to LHVs, a quantity for the total energy potential (GJ) considering complete pyrolysis of the straw tonnage from the farm can be calculated as seen in Fig. 7. Running the system at 900 °C and 5 rpm was the optimum condition to maximise the energy content of the syngas. This came down to the greater impact increasing temperature had on the increase in volume, compared to the decreasing LHV effects. Beans, at 900 °C and 10 rpm, were an exception to this rule due to the significant decrease in LHV for 5 rpm, corresponding to a high peak in H_2_ production. A similar effect can be seen for OSR under 900 °C and 5 rpm conditions, where the volume of syngas obtained impacted the energy calculations. Peak volume occurred at 850 °C, which was in line with the energy potential.

The energy potential from the total feedstock is 3900 GJ/year. Thus, the farm’s current demand of 560 GJ/year requires only 14.4% of the syngas potential. To effectively meet the farm’s energy demand, further processing would be required so that higher quality fuels are produced, such as through Fischer–Tropsch synthesis [20], used to produce liquid fuels. Modifications to the current farming equipment would also be needed to fully utilise the energy. However, an excess potential of 85.6% allows for the efficiency of further processes and fluctuations obtained when scaling to an industrial scale. Therefore, it is feasible that there is sufficient energy within the gas to account for this, demonstrating clear promise in moving the agricultural industry towards becoming self-sufficient.

### Efficiency

Since the energy output from the syngas and the energy requirements of the system have been determined, the efficiency of the overall process can be quantified. This is displayed as cold gas efficiency (CGE), net efficiency, and electrical efficiency.

#### Cold gas efficiency

**Fig. 8:**
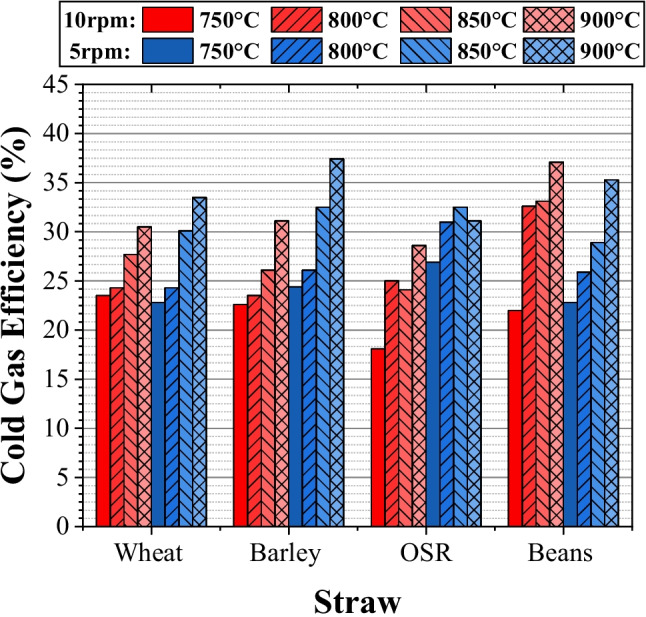
Cold gas efficiency

The results under the given conditions are shown in Fig. 8, with values ranging from 18.1 to 37%. It shows how CGE increases with temperature and decreases with an increased feed rate, which can be attributed to the effects of volume and the LHV of the syngas produced. A lack of studies focusing on the CGE of straw pyrolysis meant that comparison to similar studies was challenging. However, available CGE values quoted in the literature are significantly higher than the values presented here. Seo et al. state a range of 38 to 47% for the pyrolysis of sawdust at 900 °C [21] and Basu claims values of around 80% for various gasifier types [18]. This demonstrates the wide variance in results and indicates the individualistic and complex nature of pyrolysis, with many variables impacting the process, such as feedstock, temperature, and design.

#### Net efficiency

**Fig. 9:**
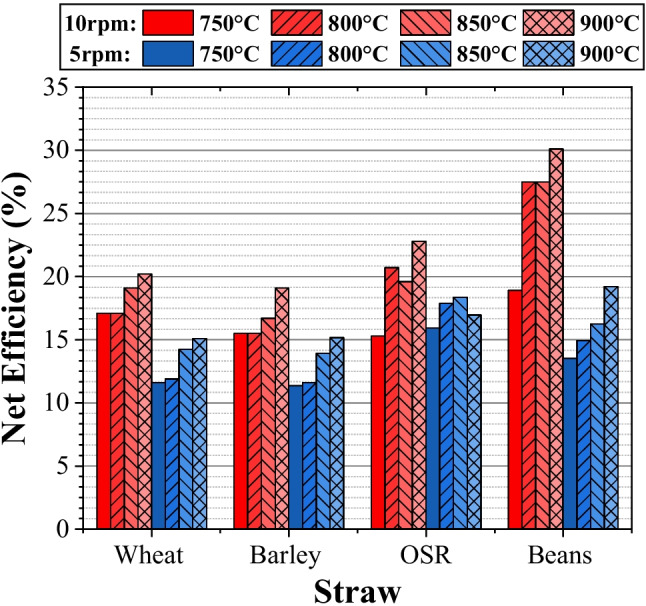
Net efficiency

The results presented in Fig. 9 are significantly lower than those presented by the CGE in Fig. 8, with the lowest being 14.6% and the highest 30.1%. This considered the run duration, which impacted the results of wheat and barley more significantly due to their higher run times per kg and meant that the system needed to be operational for significantly more time to pass the same quantity of feedstock. The benefits obtained from these feedstocks such as a higher syngas quality, higher LHV, and higher volume produced were negated by this larger energy demand, depleting their efficiencies.

The ${\eta }_{NET}$ demonstrates the low energy output in comparison to the energy required to operate the system. However, if the electrical demands of the system were met by renewable sources, it would be possible to convert green electrical sources to chemical potential, an important consideration for hard-to-electrify industries such as agriculture. An industrial system would need to be scaled almost 200 times (when considering the feed rate). If the heater power is also scaled linearly, the required power would be between 100 and 200 kW, a value easily supplied by wind and other renewables implemented by the farm [22]. Therefore, there is a potential for such a system to be applied from an energy perspective. There would be issues surrounding the scaling of the system from the 1 kg/h (biomass) scale currently in place, as well as the huge costs involved in the application of such a system, which would impact the economic feasibility.

#### Electrical efficiency

A final point of interest assumes that straw is a waste product, as it is not the primary product of the crop. This is valid based on a closed-loop, circular economy approach. If feedstock input energy was neglected due to it not constituting useful energy, a new efficiency termed electrical efficiency can be determined considering the useful energy required to operate the system and the quantity of syngas produced.

**Fig. 10:**
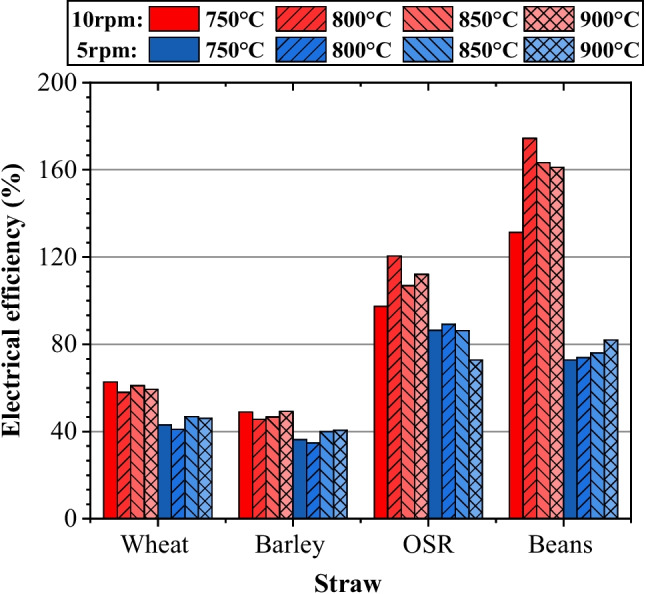
Electrical efficiency

As illustrated in Fig. 10, for OSR and beans, electrical efficiency values greater than 100% were consistently obtained for 10 rpm conditions, though efficiencies greater than 100% are only possible by solely considering the useful energy input. Recognising this, OSR and beans could provide more energy than the electrical input. Due to the feed rate’s minimal impact on syngas quality, an argument could be put forward to run the system at greater rates of 15 rpm or 20 rpm. This could have a minor impact on the gas quality, particularly at higher temperatures, whilst still reducing the energy demand of the system. A decision needs to be made on whether to optimise the energy potential of the syngas or the efficiency of the process. Considering the case study only requires 14.4% of the current syngas (not including the efficiency of further processing and energy requirements of pre-processing), a decrease in the energy quality and volume produced by running at a higher speed would be beneficial if it reduces the electrical input requirements.

Pre-processing techniques to improve the density of the feedstock, increasing the throughput of the system, and decreasing the total electrical demand would be beneficial. Pelletised straw is a common practice and there have been significant studies reporting benefits, such as a mass feed rate of 5 kg/h for barley straw, with an efficiency of more than 73% [23]. Therefore, additional studies to assess the effect of pelletised straws and their effect on syngas composition, volume, and total efficiency are recommended.

### Statistical analysis

This study investigates a range of feedstocks, temperatures, and feed rates to determine the optimal configuration for maximising energy content. The analysis evaluates cold gas, net, and electrical efficiencies. Statistical analysis, conducted using Minitab software, is employed to assess the validity of the results, providing confidence in the reliability of the conclusions drawn in this study.

#### General linear model: CGE versus feed rate and temperature

This section presents the fitting of a general linear model to the experimental data, with cold gas efficiency as the response variable and feed rate and temperature as the determining factors. This methodology facilitated a straightforward statistical analysis. The resulting R^2^ values for wheat, barley, OSR, and beans are 95.54%, 95.33%, 92.00%, and 93.16%, respectively.

The R^2^ values obtained for all feedstocks exceed 90%, indicating a high degree of accuracy in the data [24]. Reporting the fit of the statistical model through the R^2^ value facilitates comparisons between models with the same number of predictors, thus providing a clear basis for comparing the data presented in this study. The regression equations for each of the feedstocks considered are presented below. They consist of values related to feed rate (FR5 and FR10) and temperature (T750, T800, T850, and T900).

For wheat:

4 $$CGE=27.09+0.59FR5-0.59FR10-3.94T750-2.79T800+1.81T850+4.91T900$$

For barley:

5 $$CGE=27.96+2.14FR5-2.14FR10-4.46T750-3.16T800+1.34T850+6.29T900$$

For OSR:

6 $$CGE=27.16+3.21FR5-3.21FR10-4.66T750-0.84T800+1.14T850+2.69T900$$

For beans:

7 $$CGE=29.74-1.46FR5+1.46FR10-7.43T750-0.49T800+1.26T850+6.56T900$$

The following generic regression Eq. (8) was formulated by taking the mean of each term for all feedstock types. Although this method may exhibit reduced accuracy compared to the individual equations for each feedstock, it provides a reasonable estimate of the expected outcomes when applying different biomasses.

General regression equation for CGE:

8 $$CGE=27.99+1.12FR5-1.12FR10-5.01T750-1.40T800+1.39T850+5.11T900$$

#### General linear model: NET efficiency versus feed rate and temperature

By setting net efficiency as the response variable and rerunning the general linear model, a statistical analysis was conducted to evaluate the net efficiency of the pyrolysis process. The R^2^ results for wheat, barley, OSR, and beans are 97.05%, 94.5%, 77.95%, and 92.18%, respectively. These values indicate a strong correlation between the model and the observed data, suggesting that the model accurately represents the underlying relationships for each feedstock. The corresponding regression equations for NET are provided below.

For wheat:

9 $$CGE=17.68-0.70FR5+0.70FR10-1.68T750-1.48T800+1.03T850+2.13T900$$

For barley:

10 $$CGE=16.71+0.01FR5-0.01FR10-1.66T750-1.51T800+0.59T850+2.59T900$$

For OSR:

11 $$CGE=20.91+1.31FR5-1.31FR10-3.01T750+0.94T800+0.69T850+1.39T900$$

For beans:

12 $$CGE=23.28-2.73FR5+2.73FR10-5.13T750+0.08T800+0.92T850+4.13T900$$

Equation (13) is formulated by averaging the terms across the four feedstocks, resulting in a generalized form.

General regression equation for NET:

13 $$CGE=19.64-0.53FR5+0.53FR10-2.87T750-0.49T800+0.81T850+2.56T900$$

#### General linear model: electrical efficiency versus feed rate and temperature

This section outlines a simple statistical analysis conducted by setting electrical efficiency as the response variable. The analysis evaluates the R^2^ value and presents the corresponding regression equations for each feedstock type. The experimental data for various feedstocks was analysed using a general linear model, yielding the R^2^ values of wheat (R^2^ = 97.76%), barley (R^2^ = 95.37%), OSR (R^2^ = 86.98%), and beans (R^2^ = 96.77%) for electrical efficiency. These values demonstrate a high degree of correlation between the model and the observed data, indicating that the model effectively captures the underlying relationships within the data for each feedstock. The regression equations for electrical efficiency are as follows:

For wheat:

14 $$CGE=52.26-8.04FR5+8.04FR10+0.59T750-2.76T800+1.74T850+0.44T900$$

For barley:

15 $$CGE=42.76-4.86FR5+4.86FR10-0.11T750-2.61T800+0.59T850+2.14T900$$

For OSR:

16 $$CGE=96.46-12.79FR5+12.79FR10-4.51T750+8.39T800+0.14T850-4.01T900$$

For beans:

17 $$CGE=116.87-40.70FR5+40.70FR10-14.78T750+7.28T800+2.77T850+4.72T900$$

The general equation presented in Eq. (18) is an accumulation of the individual equations for each feedstock type and therefore presents a relationship between feed rate, temperature, and electrical efficiency that can be applied to any feedstock.

General regression equation for electrical efficiency:

18 $$CGE=77.09-16.60FR5+16.60FR10-4.70T750-2.58T800+1.31T850+0.82T900$$

## Conclusion

This manuscript verifies the clear potential for applying an electrically heated, continuous auger pyrolyser to support the agricultural industry in becoming self-sufficient. It assesses how syngas composition, volume, and LHV vary with pyrolysis conditions, allowing for the calculation of the energy potential and pyrolysis efficiency by considering the flow rate recorded during each experiment. A case study of a medium-scale arable farm provided the origin for this study, supplying feedstocks and its energy requirements. This provided a basis for comparison, allowing for a closed-loop circular economy assessment.

Key points include the following:

- Greater temperatures and lower feed rates resulted in a higher volume parentage of CO and H_2_ due to greater degradation of the complex molecules.
- Components from the ultimate, proximate, and lignocellulosic analysis impacted the syngas composition, shifting the superposition of separate reactions accordingly.
- The maximum energy content possible from the syngas occurred when the pyrolyser operated at 900 °C and 5 rpm due to the greater volume of gas produced, outweighing the reduced LHV obtained.
- Increased temperatures and reduced feed rates resulted in higher values for the cold gas and net efficiencies with maximum values of 37.1% and 30.1%, respectively.
- Electrical conversion efficiency varied due to the high impact of feed rate on run time and thus the electrical demand placed on the system.
- From this, a maximum energy potential of 3900 GJ/year is contained within the syngas with the farm’s demand of 560 GJ/year resulting in a requirement of 14.4% to meet the needs of the case study. This demonstrates the opportunity of using an electrically heated, continuous auger pyrolyser for moving the agricultural industry towards net zero as there is a syngas excess of 85.6%.
- Based on the statistical analysis, a high level of predictability can be attributed to the data sets for wheat, barley, bean, and OSR feedstocks. A general linear model using CGE, NET, and electrical efficiency as the response variables, with feed rate and temperature as factors, produced high accuracy for all feedstocks.

Further work must consider issues relating to scaling up this technology to feasibly process the straw tonnage within a suitable period and realise appropriate pre-processing technologies. To improve the efficiency of the process, studies to assess the impact of higher feed rates on the energy content should be conducted with an investigation of further processes to improve the usability of the syngas. In addition, future research must address the scalability of this technology, with an emphasis on its techno-economic feasibility and potential for real-world application.
